# Characteristics of Hospitalized Rhinovirus-Associated Community-Acquired Pneumonia in Children, Finland, 2003–2014

**DOI:** 10.3389/fmed.2019.00235

**Published:** 2019-10-22

**Authors:** Maria Hartiala, Elina Lahti, Ville Forsström, Tytti Vuorinen, Olli Ruuskanen, Ville Peltola

**Affiliations:** ^1^Department of Pediatrics and Adolescent Medicine, Turku University Hospital, University of Turku, Turku, Finland; ^2^Child and Adolescent Clinic, City of Turku Welfare Division, Turku, Finland; ^3^Department of Virology and Clinical Virology, Turku University Hospital, University of Turku, Turku, Finland

**Keywords:** children, pneumonia, respiratory tract infection, rhinovirus, white blood cell count

## Abstract

**Background:** Rhinovirus (RV) is the most common cause of respiratory tract infections in children but, still, the clinical characteristics of RV-associated pneumonia have not been sufficiently investigated.

**Methods:** We identified children and adolescents younger than 18 years of age treated for community-acquired pneumonia as inpatients at the Turku University Hospital from 2003 to 2014 and analyzed for RV by PCR of a respiratory tract specimen. We collected the data from medical records and compared RV-positive children with RV-negative children.

**Results:** Of the study population of 313 children with pneumonia who were studied for RV, it was detected in 82 (26%). RV-positive children were younger (median age 2.6 years, interquartile range [IQR] 1.1–4.6 vs. 3.5 years, IQR 1.7–8.3, *p* = 0.002) and they had more often a history of preterm birth (16% vs. 5%, adjusted odds ratio 2.89, 95% confidence interval 1.21–6.92, *p* = 0.017) than RV-negative children. RV-positive children had a higher median white blood cell count than RV-negative children at presentation with pneumonia. The signs, symptoms, and severity of pneumonia were mostly similar in RV-positive and RV-negative children.

**Conclusions:** RV was frequently detected in young children hospitalized with community-acquired pneumonia. We identified premature birth as a factor associated with RV-positive pneumonia. The clinical features of pneumonia did not clearly differ between RV-positive and RV-negative children. Further studies are needed to clarify the clinical significance of detection of RV in children with pneumonia.

## Introduction

Pneumonia is a common cause of hospitalization in children. Rhinovirus (RV) is the most common cause of respiratory tract infections in children worldwide, and frequently detected in community-acquired pneumonia (CAP) (1–6). According to global estimates, 120 million episodes of pneumonia in children younger than 5 years of age are recorded annually (7). RV is detected in up to 46% of children with CAP (5, 8, 9). RVs are small non-enveloped RNA viruses classified into three species (A, B, and C) with approximately 160 different types (10, 11). Simultaneous circulation of several RV types in populations year-round explains the high frequency of RV infections.

The scientific literature on asthma exacerbation and wheezing illnesses caused by RV is extensive (12), but the clinical characteristics of RV-associated pneumonia in children have not been thoroughly investigated. RV is commonly present in mixed viral-bacterial and viral-viral infections (13) and a substantial proportion of asymptomatic children are positive for RV by PCR of upper respiratory tract specimens (14). The role of RV as the causative agent of pneumonia is unclear.

The aim of this study was to assess the risk factors for, clinical characteristics and prevalence of RV-associated pneumonia in children. We compared the medical record data of RV-positive and RV-negative children hospitalized with CAP.

## Methods

### Participants and Data Collection

This retrospective study involved children and adolescents younger than 18 years of age treated as inpatients at the Department of Pediatrics and Adolescent Medicine, Turku University Hospital (Turku, Finland), during a 12-year period from 2003 to 2014. To identify children who were hospitalized with CAP, we searched the Electronic Registry of the Turku University Hospital for International Classification of Diseases (ICD-10) codes related to pneumonia (J12–18, J10.0, J11.0, J85, J86, J90) with an age limit of 18 years. Of this patient population, we identified those with a diagnostic polymerase chain reaction (PCR) test for RV performed during the hospitalization for CAP. The medical records of these children were reviewed to collect the clinical and background data. Excluding preterm birth, only currently present underlying conditions were considered. Tachypnea was defined as respiratory rate >60/min in infants younger than 2 months of age, >50/min in infants from 2 to 12 months, >40/min in children from 1 to 5 years and >30/min in children 6 years of age or older.

The study was approved by the Institutional Review Board at the Clinical Research Centre of the Turku University Hospital.

### Laboratory Detection

The diagnostic tests for RV were *in house* qualitative reverse transcription (RT) -PCR assays and commercial multiplex PCR tests for respiratory viruses including RV, which were in routine use in the diagnostic laboratory during the study period. The first *in house* PCR used detected RV and enterovirus (15). It was later replaced by a triplex test for RV, enterovirus and respiratory syncytial virus (16). The analytical procedures, sensitivities and specificities of the *in house* tests are described in the above-cited references. Since 2008 we also used commercial multiplex PCR kits, first a Seeplex RV12 Ace detection kit and since 2013 Anyplex RV16 detection kit (both from Seegene, Seoul, Korea). The commercial multiplex PCR methods may have slightly lower sensitivities for RV than the *in house* tests (17). A child was considered as a RV-positive case if RV was detected either by the in-house PCR or the multiplex PCR or both.

### Data Analysis

RV-positive children were compared with RV-negative children. To test whether the results were affected by the presence of other viruses, we conducted a sensitivity analysis of children with a sole RV finding (no other viruses detected) compared with those who had no viruses detected. Data were presented as proportions, or medians with interquartile ranges (IQR). Univariate comparisons were performed for continuous data by use of the Wilcoxon rank-sum test and for categoric data by use of the χ^2^ test or Fisher's exact test. All tests were two-sided. The significance level was *P* < 0.05. A multivariate logistic regression analysis was conducted to examine the independent risk factors for RV-positive CAP. The final model included age, sex and presence of the following prior diseases or conditions: asthma or reactive airway disease, premature birth, neurological condition, cardiovascular disease, and atopic eczema or sensitization to aeroallergen. Statistical analyses were performed using SAS system for Windows, version 9.4. (SAS Institute Inc., Cary, NC, USA) or SPSS version 23.0 (IBM SPSS Statistics, IBM Corp., Armonk, NY, USA).

## Results

### Study Population, Characteristics and Underlying Conditions

**Figure 1:**
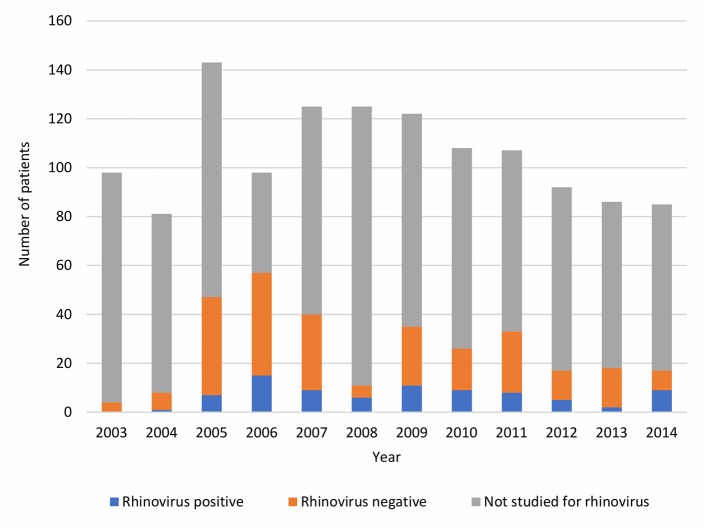
Yearly numbers of pneumonia inpatients stratified by RV status during years 2003–2014.

Of a total of 2484 children with CAP, 1270 (51%) were treated as inpatients and 1214 (49%) as outpatients. Hospitalization was needed for 81 to 143 children with CAP per year (Figure 1). Inpatients were younger than outpatients (median age 2.88 [IQR 1.49–5.63] years vs. 3.38 [1.77–7.15] years, p < 0.001). Of 1270 inpatients 313 (25%) had PCR diagnostics for RV done during the hospitalization, and 82 (26% of 313) had RV detected. Children treated as outpatients for pneumonia were not tested for RV.

**Figure 2:**
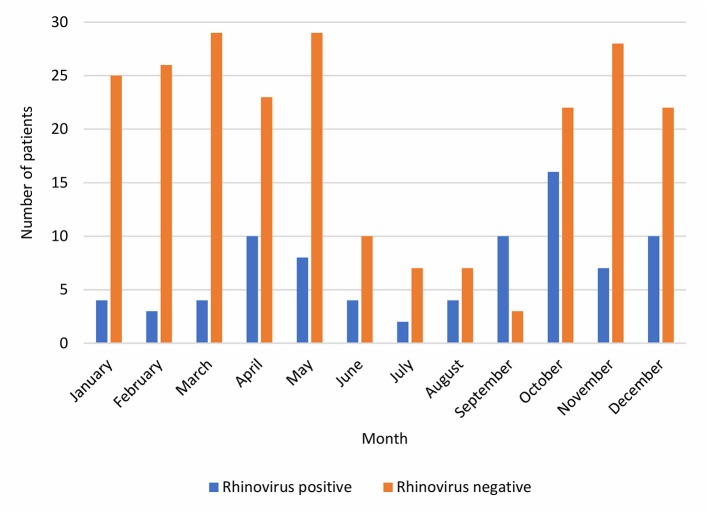
The cumulative monthly numbers of RV-positive and RV-negative pneumonia inpatients during years 2003–2014.

The final study population (*n* = 313) consisted of 171 males (55%) and 142 females (45%) with a median age of 3.09 (IQR 1.53–7.35) years (Table 1). All patients had radiologically confirmed pneumonia. The monthly peak occurrence of RV pneumonia was in October (Figure 2). RV-positive patients were younger (median age 2.59 [IQR 1.08–4.59] years) than RV-negative patients (median age 3.51 [IQR 1.68–8.26] years) (*p* = 0.002).

**Table 1:** Demographic characteristics and underlying conditions of children with community-acquired pneumonia requiring hospitalization; RV-positive patients compared to RV-negative patients.

**Demographic characteristics and underlying conditions**	**RV-positive,** ***n* = 82**	**RV-negative,** ***n* = 231**	**Univariate analysis** ***P*-value**	**Logistic regression analysis**	
				***P*-value**	**OR (95% CI)**
Age, year – median (IQR)	2.59 (1.08–4.59)	3.51 (1.68–8.26)	0.002^a^	0.010	0.91 (0.86–0.98)
Age group – no. (%)					
<2 years	33 (40)	77 (33)			
2–4 years	32 (39)	55 (24)			
5–9 years	10 (12)	53 (23)			
10–17 years	7 (9)	46 (20)			
Males – no. (%)	49 (60)	122 (53)	0.278^b^	0.241	1.38 (0.81–2.35)
Underlying condition – no. (%)	39 (48)	94 (41)	0.280^b^		
Atopic eczema or sensitization to aeroallergen	22 (27)	43 (19)	0.115^b^	0.154	1.59 (0.84–3.01)
Preterm birth	13 (16)	12 (5)	0.002^b^	0.017	2.89 (1.21–6.92)
Asthma or reactive airway disease	12 (15)	28 (12)	0.558^b^	0.363	1.45 (0.65–3.20)
Cardiovascular disease	6 (7)	8 (3)	0.209^c^	0.366	1.76 (0.52–5.97)
Neurological condition	3 (4)	11 (5)	1.000^c^	0.526	0.61 (0.14–2.78)
Malignancy or immunosuppression	1 (1)	6 (3)	0.681^c^		

*CI, confidence interval; IQR, interquartile range; OR, odds ratio; RV, rhinovirus*.

a *Wilcoxon rank-sum test*.

b *χ^2^ test*.

c *Fisher's exact test*.

Preterm birth was more frequent in RV-positive (16%) compared to RV-negative children (5%, *p* = 0.002 in univariate analysis). This association remained significant in the multivariate logistic regression analysis (OR 2.89, 95% CI 1.21–6.92, *p* = 0.017). Other underlying conditions were not significantly associated with RV-positive CAP. Fifteen percent of RV-positive and 12% of RV-negative children had a diagnosis of asthma at the time of admission. Atopic eczema or sensitization to aeroallergen was present in 27% of RV-positive and in 19% of RV-negative children.

### Clinical Findings

The clinical profiles of pneumonia were in general similar in children with or without RV, the most common symptoms being fever and cough in both groups (Table 2). However, the frequency of documented fever was lower in RV-positive than in RV-negative children (84% vs. 97%, *p* < 0.001). Dyspnea or shortness of breath was present in 35% of RV-positive patients and in 28% of RV-negative patients, and tachypnea in 30% and 29%, respectively. The duration of symptoms before referral was shorter in RV-positive (median 3 [IQR 1–7] days) than in RV-negative children (median 5 [IQR 2–8] days) (*p* = 0.006).

**Table 2:** Clinical features, laboratory results and outcomes of children with community-acquired pneumonia requiring hospitalization; RV-positive patients compared to RV-negative patients.

**Clinical features, laboratory results and outcomes**	**RV-positive,** ***n* = 82**	**RV-negative,** ***n* = 231**	***P-*value**
**Clinical features – no. (%)**			
Cough	58 (71)	185 (80)	0.081^a^
Fever	69 (84)	223 (97)	<0.001^a^
Tachypnea	25 (30)	67 (29)	0.800^a^
Dyspnea or shortness of breath	29 (35)	65 (28)	0.220^a^
Rhinitis or nasal congestion	36 (44)	83 (36)	0.201^a^
Sore throat or hoarse voice	7 (9)	13 (6)	0.355^a^
Headache	7 (9)	23 (10)	0.708^a^
Muscle pain	5 (6)	10 (4)	0.550^b^
Chest pain or abdominal pain	12 (15)	42 (18)	0.465^a^
Vomiting	24 (29)	69 (30)	0.918^a^
Otitis media	17 (21)	67 (29)	0.146^a^
Abnormal breath sounds	52 (63)	151 (65)	0.750^a^
Crackles	37 (45)	99 (43)	0.722^a^
Decreased breath sounds	17 (21)	58 (25)	0.425^a^
Wheezing	10 (12)	26 (11)	0.819^a^
Duration of symptoms before referral, days – median (IQR)	3.0 (1.0–7.0)	5.0 (2.0–8.0)	0.006
Antibiotic treatment received before referral – no. (%)	11 (13)	49 (21)	0.123^a^
**Laboratory results**			
WBC count on admission, × 10^9^/L – median (IQR)^d^	16.4 (12.2–25.0)	14.1 (8.5–20.4)	0.002^c^
Highest WBC count, × 10^9^/L – median (IQR)^e^	17.4 (13.4–25.1)	14.8 (9.5–20.6)	0.003^c^
0–3.9 × 10^9^/L – no. (%)	1 (1)	4 (2)	
4–14.9 × 10^9^/L – no. (%)	28 (35)	112 (49)	
≥15 × 10^9^/L – no. (%)	52 (64)	113 (49)	
CRP on admission, mg/L – median (IQR)^f^	79 (20–192)	87 (25–181)	0.998^c^
Highest CRP, mg/L – median (IQR)^g^	86 (28–192)	100 (30–214)	0.582^c^
<20 mg/L – no. (%)	18 (22)	44 (19)	
20–39 mg/L – no. (%)	8 (10)	25 (11)	
40–79 mg/L – no. (%)	13 (16)	30 (13)	
≥80 mg/L – no. (%)	42 (52)	131 (57)	
**Outcomes**			
Length of stay, days – median (IQR)	2.0 (1.0–4.0)	2.0 (1.0–3.0)	0.802^c^
Intensive care unit admission – no. (%)	16 (20)	35 (15)	0.358^a^
Oxygen supplementation – no. (%)	28 (34)	63 (27)	0.239^a^
Invasive mechanical ventilation – no. (%)	8 (10)	11 (5)	0.112^b^
Lung abscess, necrotising pneumonia or empyema – no. (%)	5 (6)	20 (9)	0.473^a^
Death in the hospital – no. (%)	0 (0)	1 (0.4)	

*CRP, C-reactive protein; IQR, interquartile range; RV, rhinovirus; WBC, white blood cell*.

a *χ^2^ test*.

b *Fisher's exact test*.

c *Wilcoxon rank-sum test*.

d *Data available on 80 RV-positive and 228 RV-negative children*.

e *Data available on 81 RV-positive and 229 RV-negative children*.

f *Data available on 79 RV-positive and 228 RV-negative children*.

g *Data available on 81 RV-positive and 230 RV-negative children*.

### Laboratory Results

The white blood cell (WBC) count determined on admission was higher in RV-positive than in RV-negative children (median 16.4 [IQR 12.2–25.0] × 10^9^/L vs. 14.1 [8.5–20.4] × 10^9^/L, *p* = 0.002), whereas the C-reactive protein (CRP) concentrations were not significantly different (Table 2).

At least one virus other than RV was detected by PCR in 13 (16%) of 82 children positive for RV and in 45 (19%) of 231 children negative for RV. Five children had pathogenic bacteria in the blood culture: 1 RV-positive and 3 RV-negative children had *Streptococcus pneumoniae* and 1 RV-negative child had *Streptococcus pyogenes* bacteremia.

### Treatment and Outcome

Thirteen percent of RV-positive and 21% of RV- negative patients received antimicrobial therapy before referral to the hospital, and all patients received antimicrobial therapy during hospitalization. Of RV-positive children, 34% received oxygen supplementation, 20% were admitted to the intensive care unit, and 10% required invasive mechanical ventilation, whereas the corresponding rates were, respectively, in RV-negative children 27, 15, and 5%. These differences were not statistically significant. The median duration of hospitalization was 2.0 days in both groups. Complicated pneumonia (defined as lung abscess, necrotising pneumonia, or empyema) was documented in 5 (6%) of RV-positive and in 20 (9%) of RV-negative patients. A 1-year-old boy with no underlying conditions, negative for RV but positive for influenza B and adenovirus, died of pneumonia after 5 days of hospital treatment. Thus, the mortality rate in the study population was 0.3%.

### Sensitivity Analysis

As a test of sensitivity of our results to effects of other viruses, we compared children with a sole RV finding (no other virus detected) (*n* = 69) with those who had no virus detected (*n* = 186). The findings remained essentially similar. Children with RV only were younger than virus-negative children (median age 2.92 [IQR 1.11–4.67] vs. 4.52 [1.81–9.16] years, *p* = 0.002) and they had more often a history of preterm birth (14% vs. 5%, univariate *p* = 0.009, multivariate analysis OR 2.98 [95% CI 1.10–8.05], *p* = 0.032). Similar to results of all subjects, the duration of symptoms before referral was shorter, fever was documented less frequently, and the median WBC count was higher in sole RV-positive children compared to virus-negative children.

## Discussion

In this study covering a 12-year period, RV was detected in 26% of children hospitalized with radiologically-confirmed CAP in whom testing was performed. RV-associated CAP was particularly common in young children and in children born prematurely. Clinically, RV-associated pneumonia did not clearly differ from RV-negative pneumonia. CRP and WBC levels were in most cases high and patients were treated with antibiotics because of suspected bacterial pneumonia.

Hospitalization was required in about half of all children evaluated at the emergency department for CAP, which is similar to earlier data from Finland (18, 19). The yearly number of children hospitalized with CAP was variable and had a downward trend during the latter part of the study period (Figure 1). Worldwide, introduction of pneumococcal conjugate vaccines has had a substantial impact on children's hospitalizations with all-cause pneumonia and, also, on hospitalizations with virus-positive pneumonia (20). In Finland, a 10-valent pneumococcal conjugate vaccine was included in the national immunization program for all children in 2010. As pneumococcal pneumonia cases decrease due to vaccinations, viruses are presumably becoming even more substantial cause of pneumonia. This was not seen for RV in this study, which did not include a long period of time after introduction of pneumococcal vaccinations; the yearly proportion of RV-positive CAP of those tested for RV did not systematically change. The RV prevalence documented in our study is in concordance with previous literature reporting the detection rate of RV from 14 to 46% in children hospitalized with pneumonia (2, 3, 5, 8, 9, 13). RV infections occur year-round but most commonly during autumn and spring (21), which was seen also in our study.

In our study, RV-positive pneumonia patients were younger than RV-negative patients. Children with RV-positive and RV-negative pneumonia were largely similar in respect of the severity of illness and the response to treatment. The need of oxygen supplementation, treatment at the intensive care unit, and invasive mechanical ventilation were non-significantly more common in those infected with RV. We found only a few earlier studies considering the clinical characteristics of RV pneumonia in children. Annamalay et al. compared clinical features as well as laboratory and microbiology findings of RV-positive and RV-negative children hospitalized with pneumonia in Mozambique, without finding any significant between-group differences (22). In the study of Ahn et al., RV-positive children hospitalized for acute lower respiratory tract infections were younger, had shorter fever duration, and higher frequencies of chest retraction and wheezing than RV-negative children (23). Other studies compared children with CAP or with an unspecified lower respiratory tract infection caused by different RV types and found only marginal differences (23–25).

Wheezing illnesses (bronchiolitis, recurrent wheezing, or exacerbation of asthma) often associate with RV (11, 12, 23, 26–28). Our study focused on pneumonia, which may be sometimes difficult to differentiate from wheezing illnesses. Furthermore, pneumonia and wheezing may have common risk factors. An association between childhood pneumonia and asthma has been noticed for long (29–31). Parental asthma, earlier wheezing, and exposure to tobacco smoke are associated with more severe clinical course of pneumonia (32). In our study, asthma was slightly more common in RV-positive patients than others, but, contrary to our expectation, wheezing in auscultation of lungs was not more prevalent among RV-positive than RV-negative CAP patients. The clinical picture of RV-associated pneumonia seems to be clearly different from RV-associated wheezing illness.

We found that RV-positive patients were considerably more often prematurely born than others. Accordingly, in the study of Miller et al., prematurely born children were found to be particularly susceptible to RV infections (33). Kennedy et al. have highlighted that it is the nature and extent of the immune response to the virus that determines the symptom profile (34). Numerous pathophysiological mechanisms, such as diminished immune responses and lung function but also inflammatory and airway re-modeling pathways activated by viruses, are proposed to influence the increased risk of respiratory disorders, including RV infections, following preterm birth (35).

The role of RV as a true pneumonia pathogen is unclear (36–41). Life-threatening cases of pneumonia caused by only RV have been reported (42), and RV has been detected directly from the lung tissue from a child with pneumonia (43). RV viremia is particularly common in CAP patients with RV-C (44, 45). In our study, as well as in most other studies concerning the etiology of pneumonia, RV was detected by PCR from upper respiratory tract specimens, and the presence of the virus at the site of infection, the lung, remains unknown. As RV is frequently (approximately in 15%) detected also in asymptomatic children (14), its role as a causative agent vs. a bystander in children with pneumonia can be questioned. It should be noted, however, that the mean virus shedding time after RV infection is as short as 11 days in immunocompetent children (46). Persistent shedding of RV is not known to occur in otherwise healthy subjects. The detection of RV in asymptomatic children may reflect previous infection, ongoing asymptomatic or mild infection (possibly by less virulent RV types), or an incubation period preceding the onset of symptoms.

The identification of RV directly from the lung is not feasible in common clinical settings. In severe pneumonia or in immunocompromised subjects, obtaining invasive samples like bronchoalveolar lavage for microbiological evaluation including RV PCR would be critically informative (47). RV loads in upper respiratory tract specimens do not clearly correlate with the clinical course of the infection. Children with wheezing or only rhinitis have been reported to have similar RV loads in their nasal washes (28). On the contrary, in a recent study, higher RV viral load was associated with more severe respiratory symptoms (48). Serologic assays for RV infection could help to prove acute infections but such tests are not in routine use. Recently, despite high phylogenetic diversity of RV, the development of RV species-specific antibody test has been successful (49). Integrating studies of host response, microbe detection, and airway microbiome is a modern approach in pneumonia diagnostics (50). Development of effective vaccines and drug treatments for RV would ultimately make it possible to show the impact of RV on childhood pneumonia. Recently, Toll-like receptor 3 blockage has been studied as one of the possible options for treatment (51).

RV has been associated with a severe course of pneumonia in children, similar to our findings (52). Children with RV-positive pneumonia may have a concomitant bacterial pneumonia, or a secondary bacterial pneumonia following a RV infection. In our children with RV-associated CAP, CRP and WBC levels were elevated, and the median WBC counts were even higher in the RV-positive than in the RV-negative group. Increased levels of WBC have been earlier reported in RV-related lower respiratory tract infections (53). CRP and WBC are not highly specific in differentiation between viral and bacterial infections and better biomarkers or microbiologic methods would be needed in order to confirm or exclude bacterial co-infection in RV-associated CAP. Mixed infections are increasingly recognized and possibly associated with a more severe course of pneumonia, particularly the combination of RV and *S. pneumoniae* (13, 54–57). *In vitro*, RV infection has been shown to stimulate adhesion of *S. pneumoniae* to airway epithelial cells via increases in the platelet-activating factor receptors (58). The impairment of immune response to bacterial products and phagocytosis of bacteria in human macrophages in response to RV exposure has been documented (59, 60). Moreover, the seasonality of pneumonia coinciding with viral lower respiratory tract infections has been observed (61).

In the absence of effective antiviral drugs for other respiratory viruses than influenza A and B virus, the clinical significance of diagnosing viral etiology of pneumonia has not been firmly established. Avoidance of the unnecessary use of antibiotics and excessive laboratory or other tests, shortening of the length of stay in the hospital, prediction of the clinical course of illness, and prevention of transmission to other patients by isolation or other methods are potential benefits of virus detection, in addition to surveillance of local epidemiology of seasonal viruses. In our study, the characteristics of RV-associated pneumonia were closely similar to RV-negative pneumonia, and identification of RV did not result in the withholding of antibiotic treatment. The matter could be different in milder pneumonia cases not needing hospitalization.

Our study has notable limitations related to the retrospective setting. First, our study population was somewhat selected as RV tests were not routinely performed for all CAP patients. However, our study population can be considered to be a representative sample of overall CAP inpatients as key figures in patients undergoing RV detection in the present study and in CAP inpatients in our previous prospective CAP study are comparable (13). Second, data on the types of RV and viral loads were not available, and other viruses and bacteria were not comprehensively analyzed. In our previous CAP study with induced sputum as a diagnostic sample and patient population overlapping with our present study, 64% of RV findings belonged to RV A species and 36% to RV C species, and viral-bacterial co-infections were frequent (13). Similarly, other researchers have reported that RV A and C species are frequent and RV B species rare in children with pneumonia (23, 24). Third, the clinical data was collected from the medical records, which might be incomplete. Fourth, diagnostic methods and clinical practices varied during the 12-year study period. Fifth, our study included only hospitalized CAP patients. Sixth, the lack of a control group of healthy children is an obvious limitation in this study.

In conclusion, RV is frequently present in childhood pneumonia, particularly in vulnerable patients such as young children and those with a history of preterm birth. Among children hospitalized with CAP in this study, RV-positive pneumonia was a rather severe disease with high levels of inflammatory biomarkers and a clinical course that often necessitated intensive care, with no justification for withholding antibiotic treatment. Other studies are needed to establish the clinical characteristics of RV pneumonia in outpatients. Considering the large global burden of pneumonia and the high prevalence of RV in children with pneumonia, development of a diagnostic marker indicating RV as a true cause of the disease, and drugs and vaccines for RV-specific treatment and prevention could have major significance in the future.

## Data Availability Statement

The datasets for this manuscript are not publicly available because: Participant privacy prevents public sharing of individual-level data. Requests to access the datasets should be directed to Maria Hartiala, mkhonk@utu.fi.

## Ethics Statement

This study involved only retrospective review of medical record data that had been collected during routine patient care. The study was approved by the Institutional Review Board at the Clinical Research Centre of the Turku University Hospital with a statement that an evaluation by the Ethics Committee was not needed.

## Author Contributions

MH and VF collected the clinical data. TV was responsible for virus diagnostics. MH analyzed data and wrote the manuscript with support of VP, EL, VF, TV, and OR. VP supervised the project.

### Conflict of Interest

The authors declare that the research was conducted in the absence of any commercial or financial relationships that could be construed as a potential conflict of interest.
